# Supplementary material for: Elevated miR-29c-5p Expression in Nipple Aspirate Fluid Is Associated with Extremely High Mammographic Breast Density
Source: Cancers (Basel). 2022 Aug 5;14(15):3805. doi: 10.3390/cancers14153805 (PMC9367509; doi:10.3390/cancers14153805)
Supplement: Supplementary file 1 [file cancers-14-03805-s001.zip › cancers-1839166-supplementary.pdf]

Supplementary Material

Table of Contents

Supplementary Figure S1: Logistic regression analysis with the four candidate differentially expressed human mature miRNAs in the discovery cohort—page 2

Supplementary Figure S2: Unsupervised hierarchical clustering of 41 nipple aspirate fluid samples based on the expression pattern (delta CT) of four miRNAs differentially expressed between extremely high mammographic density (MD) and very low MD categories in the discovery cohort—page 3

Supplementary Figure S3: Pearson correlation analysis between microfluidics-based RT-qPCR profiling results (X axis; CRT value) and regular-volume RT-qPCR individual assay results (Y axis; CT value) (quality control for profiling) for 14 selected miRNAs showing acceptable concordance—page 4

Supplementary Table S1: List of Taqman Advanced assays present in the 754-miRNA panel used for NAF sample profiling—page 5

Supplementary Table S2: Taqman advanced miRNA assays and associated assay IDs used in the study—page 21

Supplementary Table S3: Raw CT values for 4 candidate miRNAs and endogenous control miRNA hsa-miR-125a-5p in the validation cohort—page 22

Supplementary Table S4: Baseline table comparing the discovery cohort (N=41) with the validation cohort (N = 170)—page 26

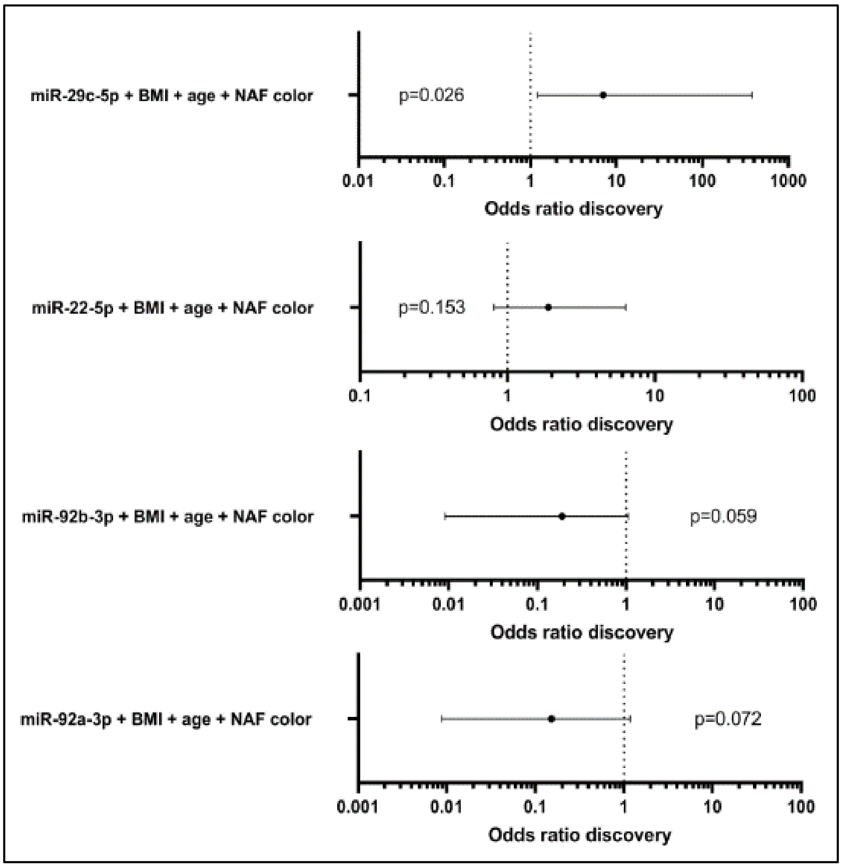

Figure S1. Logistic regression analysis with the four candidates differentially expressed human mature miRNAs in the discovery cohort. Odds ratios (OR) from the logistic regression analysis including the four human miRNAs are depicted. MiRNAs with P-values < 0.2 were considered of interest for subsequent validation. MiR-92a-3p (OR=0.153 (95% CI 0.01-1.18); p=0.072) and miR-92b-3p (OR=0.19 (95% CI 0.01-1.06); p=0.059) were negative predictors for extremely high MD (down-regulated versus very low MD), whereas miR-22-5p (OR=1.90 (95% CI 0.80-6.31); p=0.153) and miR-29c-5p (OR=7.02 (95% CI 1.21-380.67); p=0.026) were positive predictors for extremely high MD (up-regulated versus very low MD).

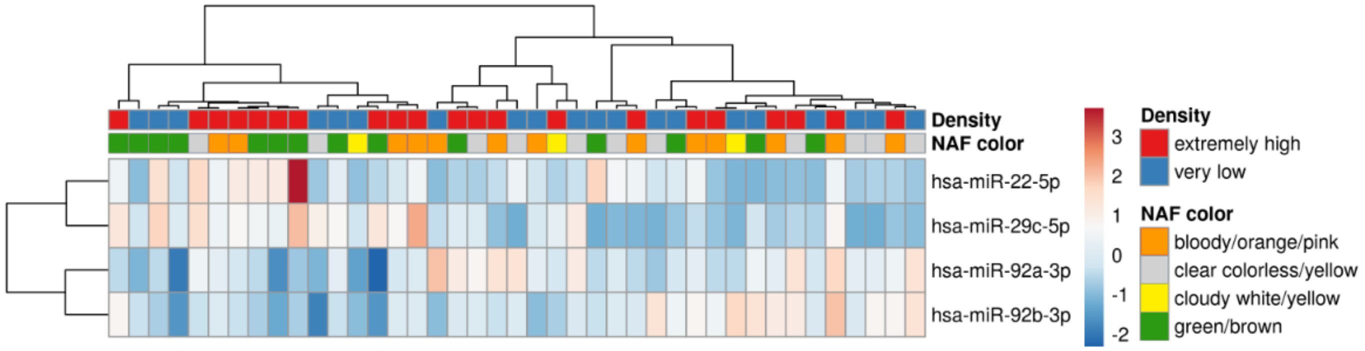

Figure S2. Unsupervised hierarchical clustering of 41 nipple aspirate fluid samples based on the expression pattern (delta CT) of four miRNAs differentially expressed between extremely high mammographic density (MD) and very low MD categories in the discovery cohort. Rows are centred; unit variance scaling is applied to rows. Imputation was used for missing value estimation. Both rows and columns are clustered using correlation distance and average linkage. The heatmap scale represents 2^ -delta CT values with low DCT (red) indicating high miRNA expression and high DCT (blue) indicating low miRNA expression.

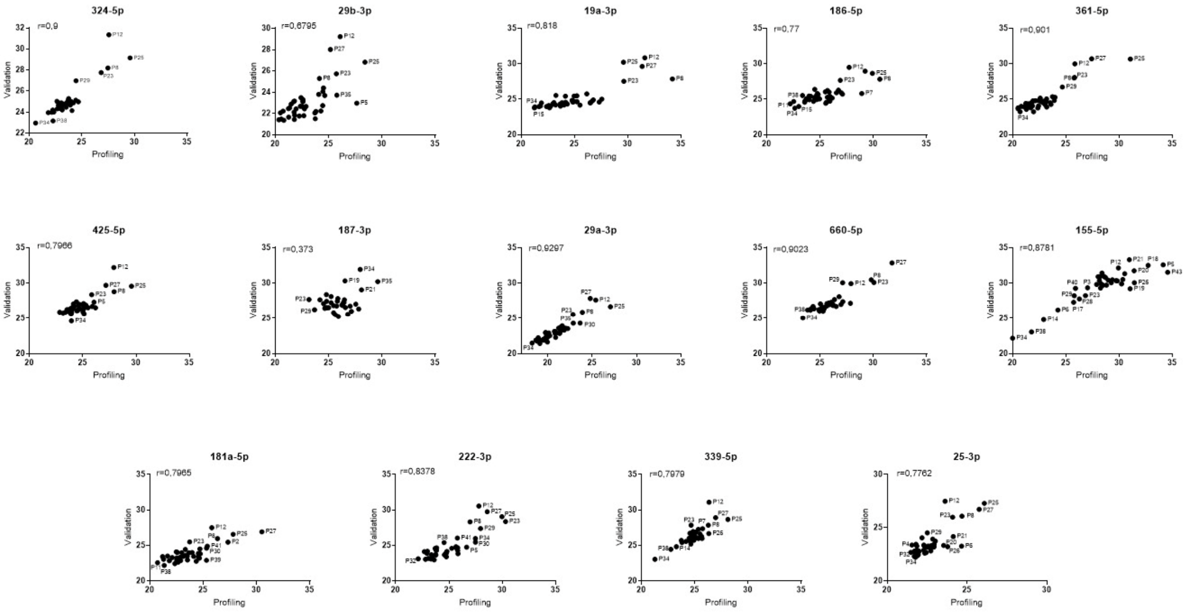

Figure S3. Pearson correlation analysis between microfluidics-based RT-qPCR profiling results (X axis; CRT value) and regular-volume RT-qPCR individual assay results (Y axis; CT value) (quality control for profiling) for 14 selected miRNAs showing acceptable concordance. Each dot represents a measurement of a NAF sample (N=41) by both methods.

Table S1. List of Taqman Advanced assays present in the 754-miRNA panel used for NAF sample profiling. Excel file available on the website of Thermo Fisher, titled "TaqMan OpenArray Human Advanced MicroRNA Panel Assay List" - version 1 layout.

OA position	Advanced AssayID	Advanced Assay Name
**A1a2**	478872_mir	hsa-miR-409-5p
**A1a3**	478092_mir	hsa-miR-424-5p
**A1a4**	478007_mir	hsa-miR-30b-5p
**A1a5**	478587_mir	hsa-miR-29a-3p
**A1a6**	478125_mir	hsa-miR-485-3p
**A1a7**	478308_mir	hsa-miR-484
**A1a8**	477854_mir	hsa-miR-380-3p
**A1b1**	478826_mir	hsa-miR-323b-5p
**A1b2**	478126_mir	hsa-miR-485-5p
**A1b3**	478293_mir	cel-miR-39-3p
**A1b4**	478561_mir	hsa-miR-449a
**A1b5**	478591_mir	hsa-miR-302b-3p
**A1b6**	478086_mir	hsa-miR-411-5p
**A1b7**	478024_mir	hsa-miR-324-5p
**A1b8**	478008_mir	hsa-miR-30c-5p
**A1c1**	477816_mir	hsa-miR-381-3p
**A1c2**	478860_mir	hsa-miR-376b-3p
**A1c3**	478790_mir	hsa-miR-296-3p
**A1c4**	478087_mir	hsa-miR-412-3p
**A1c5**	477849_mir	hsa-miR-429
**A1c6**	478106_mir	hsa-miR-450a-5p
**A1c7**	478113_mir	hsa-miR-455-5p
**A1c8**	478509_mir	hsa-miR-302c-3p
**A1d1**	478491_mir	hsa-miR-204-5p
**A1d2**	478411_mir	ath-miR159a
**A1d3**	477916_mir	hsa-miR-145-5p
**A1d4**	477925_mir	hsa-miR-154-5p
**A1d5**	477900_mir	hsa-miR-132-3p
**A1d6**	477908_mir	hsa-miR-140-3p
**A1d7**	478789_mir	hsa-miR-27b-5p
**A1d8**	477858_mir	hsa-miR-15a-5p
**A1e1**	478399_mir	hsa-miR-146a-5p
**A1e2**	477824_mir	hsa-miR-148b-3p
**A1e3**	477889_mir	hsa-miR-127-3p
**A1e4**	477857_mir	hsa-miR-181a-5p
**A1e5**	478312_mir	hsa-miR-139-5p
**A1e6**	477911_mir	hsa-miR-142-5p
**A1e7**	477891_mir	hsa-miR-127-5p
**A1e8**	477840_mir	hsa-miR-130b-3p
**A1f1**	477935_mir	hsa-miR-182-5p
**A1f2**	477918_mir	hsa-miR-150-5p
**A1f3**	477896_mir	hsa-miR-129-5p
**A1f4**	478624_mir	hsa-miR-10a-3p
**A1f5**	478584_mir	hsa-miR-18b-5p
**A1f6**	478313_mir	hsa-miR-15b-5p
**A1f7**	477938_mir	hsa-miR-184
**A1f8**	478551_mir	hsa-miR-18a-5p

**A1g3**	479537_mir	hsa-miR-548c-3p
**A1g4**	478166_mir	hsa-miR-582-5p
**A1g5**	478164_mir	hsa-miR-576-3p
**A1g6**	480870_mir	hsa-miR-548d-5p
**A1h3**	477860_mir	hsa-miR-16-5p
A2a2	478792_mir	hsa-miR-299-3p
**A2a3**	478000_mir	hsa-miR-28-5p
**A2a4**	478594_mir	hsa-miR-320a
**A2a5**	478459_mir	hsa-miR-376c-3p
**A2a6**	478090_mir	hsa-miR-423-5p
**A2a7**	478481_mir	hsa-miR-422a
**A2a8**	478075_mir	hsa-miR-377-3p
**A2b1**	478793_mir	hsa-miR-299-5p
**A2b2**	477974_mir	hsa-miR-214-3p
**A2b3**	478293_mir	cel-miR-39-3p
**A2b4**	478133_mir	hsa-miR-492
**A2b5**	478432_mir	hsa-miR-483-5p
**A2b6**	478578_mir	hsa-let-7f-5p
**A2b7**	478579_mir	hsa-let-7e-5p
**A2b8**	478131_mir	hsa-miR-490-3p
**A2c1**	477980_mir	hsa-miR-219a-5p
**A2c2**	478602_mir	hsa-miR-23b-3p
**A2c3**	479235_mir	hsa-miR-30e-5p
**A2c4**	478447_mir	hsa-miR-17-5p
**A2c5**	478580_mir	hsa-let-7g-5p
**A2c6**	477976_mir	hsa-miR-216a-5p
**A2c7**	477978_mir	hsa-miR-219a-1-3p
**A2c8**	478375_mir	hsa-let-7i-5p
**A2d1**	477940_mir	hsa-miR-186-5p
**A2d2**	478411_mir	ath-miR159a
**A2d3**	479189_mir	hsa-miR-887-3p
**A2d4**	478200_mir	hsa-miR-744-5p
**A2d5**	477883_mir	hsa-miR-125a-3p
**A2d6**	479134_mir	hsa-miR-653-5p
**A2d7**	477987_mir	hsa-miR-22-5p
**A2d8**	479196_mir	hsa-miR-891a-5p
**A2e1**	479121_mir	hsa-miR-642a-5p
**A2e2**	478191_mir	hsa-miR-655-3p
**A2e3**	478519_mir	hsa-miR-99a-5p
**A2e4**	478343_mir	hsa-miR-99b-5p
**A2e5**	479131_mir	hsa-miR-651-5p
**A2e6**	478207_mir	hsa-miR-885-5p
**A2e7**	478185_mir	hsa-miR-636
**A2e8**	479197_mir	hsa-miR-891b
**A2f1**	479184_mir	hsa-miR-875-3p
**A2f2**	479371_mir	hsa-miR-892a
**A2f3**	478513_mir	hsa-miR-146b-5p
**A2f4**	478785_mir	hsa-miR-24-2-5p
**A2f5**	478205_mir	hsa-miR-874-3p
**A2f6**	479186_mir	hsa-miR-876-3p
**A2f7**	479517_mir	hsa-miR-181d-5p

**A2f8**	478036_mir	hsa-miR-337-5p
**A2g3**	478027_mir	hsa-miR-326
**A2g4**	477806_mir	hsa-miR-208b-3p
**A2g5**	478389_mir	hsa-miR-374b-5p
**A2g6**	478590_mir	hsa-miR-98-5p
**A2h3**	477860_mir	hsa-miR-16-5p
**A3a2**	478076_mir	hsa-miR-378a-5p
**A3a3**	478855_mir	hsa-miR-374a-3p
**A3a4**	478091_mir	hsa-miR-424-3p
**A3a5**	478084_mir	hsa-miR-409-3p
**A3a6**	478002_mir	hsa-miR-29a-5p
**A3a7**	479362_mir	hsa-miR-30d-3p
**A3a8**	478388_mir	hsa-miR-30e-3p
**A3b1**	479448_mir	hsa-miR-30a-5p
**A3b2**	478859_mir	hsa-miR-376a-5p
**A3b3**	478293_mir	cel-miR-39-3p
**A3b4**	478799_mir	hsa-miR-302b-5p
**A3b5**	478237_mir	hsa-miR-302d-3p
**A3b6**	479401_mir	hsa-miR-30c-2-3p
**A3b7**	478012_mir	hsa-miR-31-3p
**A3b8**	478888_mir	hsa-miR-431-3p
**A3c1**	478800_mir	hsa-miR-302c-5p
**A3c2**	478775_mir	hsa-miR-218-2-3p
**A3c3**	478101_mir	hsa-miR-432-5p
**A3c4**	478311_mir	hsa-miR-138-1-3p
**A3c5**	478801_mir	hsa-miR-302d-5p
**A3c6**	479281_mir	hsa-let-7e-3p
**A3c7**	478939_mir	hsa-miR-488-5p
**A3c8**	478782_mir	hsa-miR-23a-5p
**A3d1**	478476_mir	hsa-miR-1260a
**A3d2**	478411_mir	ath-miR159a
**A3d3**	477948_mir	hsa-miR-190b
**A3d4**	478642_mir	hsa-miR-1227-3p
**A3d5**	478663_mir	hsa-miR-1256
**A3d6**	478655_mir	hsa-miR-1250-5p
**A3d7**	477998_mir	hsa-miR-27a-5p
**A3d8**	478634_mir	hsa-miR-1205
**A3e1**	477877_mir	hsa-miR-1233-3p
**A3e2**	478633_mir	hsa-miR-1204
**A3e3**	477888_mir	hsa-miR-126-5p
**A3e4**	477882_mir	hsa-miR-1247-5p
**A3e5**	478669_mir	hsa-miR-1263
**A3e6**	478654_mir	hsa-miR-1249-3p
**A3e7**	478635_mir	hsa-miR-1206
**A3e8**	478666_mir	hsa-miR-125b-2-3p
**A3f1**	478652_mir	hsa-miR-1245a
**A3f2**	478186_mir	hsa-miR-637
**A3f3**	478651_mir	hsa-miR-1244
**A3f4**	478047_mir	hsa-miR-34a-3p
**A3f5**	478668_mir	hsa-miR-1262
**A3f6**	479035_mir	hsa-miR-551b-5p

**A3f7**	478649_mir	hsa-miR-1238-3p
**A3f8**	479137_mir	hsa-miR-656-3p
**A3g3**	479045_mir	hsa-miR-559
**A3g4**	478167_mir	hsa-miR-584-5p
**A3g5**	478273_mir	hsa-miR-30a-3p
**A3g6**	478704_mir	hsa-miR-1324
**A3h3**	477860_mir	hsa-miR-16-5p
**A4a2**	477843_mir	hsa-let-7f-2-3p
**A4a3**	477866_mir	hsa-miR-106b-3p
**A4a4**	477991_mir	hsa-miR-23b-5p
**A4a5**	478778_mir	hsa-miR-221-5p
**A4a6**	478626_mir	hsa-miR-1179
**A4b1**	478619_mir	hsa-miR-100-3p
**A4b2**	479405_mir	hsa-miR-181a-3p
**A4b3**	478293_mir	cel-miR-39-3p
**A4b4**	478628_mir	hsa-miR-1182
**A4b5**	477861_mir	hsa-let-7a-3p
**A4b6**	478742_mir	hsa-miR-193b-5p
**A4c1**	478622_mir	hsa-miR-105-3p
**A4c2**	477996_mir	hsa-miR-26b-3p
**A4c3**	478620_mir	hsa-miR-101-5p
**A4c4**	477962_mir	hsa-miR-19b-1-5p
**A4c5**	477870_mir	hsa-miR-1183
**A4c6**	479162_mir	hsa-miR-708-3p
**A4d1**	479165_mir	hsa-miR-744-3p
**A4d2**	478411_mir	ath-miR159a
**A4d3**	478187_mir	hsa-miR-638
**A4d4**	479144_mir	hsa-miR-661
**A4d5**	479129_mir	hsa-miR-650
**A4d6**	478199_mir	hsa-miR-7-2-3p
**A4e1**	479126_mir	hsa-miR-647
**A4e2**	478033_mir	hsa-miR-335-3p
**A4e3**	479145_mir	hsa-miR-662
**A4e4**	479141_mir	hsa-miR-659-3p
**A4e5**	478196_mir	hsa-miR-675-5p
**A4e6**	479209_mir	hsa-miR-934
**A4f1**	478211_mir	hsa-miR-9-3p
**A4f2**	479124_mir	hsa-miR-644a
**A4f3**	479203_mir	hsa-miR-922
**A4f4**	479118_mir	hsa-miR-639
**A4f5**	478245_mir	hsa-miR-939-5p
**A4f6**	479030_mir	hsa-miR-549a
**A4g3**	479207_mir	hsa-miR-92b-5p
**A4g4**	479044_mir	hsa-miR-558
**A4g5**	479061_mir	hsa-miR-580-3p
**A4g6**	478702_mir	hsa-miR-130a-5p
**A4h3**	477860_mir	hsa-miR-16-5p
**B1a2**	478224_mir	hsa-miR-100-5p
**B1a3**	478439_mir	hsa-let-7d-5p
**B1a4**	477970_mir	hsa-miR-210-3p
**B1a5**	478773_mir	hsa-miR-217

**B1a6**	477985_mir	hsa-miR-22-3p
**B1a7**	478253_mir	hsa-miR-103a-3p
**B1a8**	478065_mir	hsa-miR-365a-3p_hsa-miR-365b-3p
**B1b1**	477971_mir	hsa-miR-2110
**B1b2**	477986_mir	hsa-miR-224-5p
**B1b3**	478293_mir	cel-miR-39-3p
**B1b4**	478056_mir	hsa-miR-361-5p
**B1b5**	478318_mir	hsa-miR-212-3p
**B1b6**	478262_mir	hsa-miR-192-5p
**B1b7**	478577_mir	hsa-let-7c-5p
**B1b8**	478044_mir	hsa-miR-342-5p
**B1c1**	478046_mir	hsa-miR-346
**B1c2**	478042_mir	hsa-miR-340-5p
**B1c3**	478366_mir	hsa-miR-345-5p
**B1c4**	478965_mir	hsa-miR-509-5p
**B1c5**	478749_mir	hsa-miR-198
**B1c6**	478316_mir	hsa-miR-203a-3p
**B1c7**	477963_mir	hsa-miR-200b-3p
**B1c8**	478417_mir	hsa-miR-202-3p
**B1d1**	478576_mir	hsa-let-7b-5p
**B1d2**	478411_mir	ath-miR159a
**B1d3**	478948_mir	hsa-miR-499a-3p
**B1d4**	478135_mir	hsa-miR-494-3p
**B1d5**	478144_mir	hsa-miR-504-5p
**B1d6**	478976_mir	hsa-miR-515-3p
**B1d7**	478203_mir	hsa-miR-769-5p
**B1d8**	479487_mir	hsa-miR-517c-3p
**B1e1**	478129_mir	hsa-miR-488-3p
**B1e2**	478148_mir	hsa-miR-518b
**B1e3**	478143_mir	hsa-miR-503-5p
**B1e4**	477909_mir	hsa-miR-140-5p
**B1e5**	478963_mir	hsa-miR-509-3-5p
**B1e6**	478954_mir	hsa-miR-502-5p
**B1e7**	478978_mir	hsa-miR-516a-5p
**B1e8**	478961_mir	hsa-miR-508-3p
**B1f1**	478981_mir	hsa-miR-518a-3p
**B1f2**	478183_mir	hsa-miR-629-5p
**B1f3**	478172_mir	hsa-miR-598-3p
**B1f4**	479219_mir	hsa-miR-943
**B1f5**	478339_mir	hsa-miR-597-5p
**B1f6**	478176_mir	hsa-miR-615-5p
**B1f7**	479108_mir	hsa-miR-624-3p
**B1f8**	479112_mir	hsa-miR-628-5p
**B1g3**	478160_mir	hsa-miR-561-3p
**B1g4**	478023_mir	hsa-miR-324-3p
**B1g5**	478213_mir	hsa-miR-95-3p
**B1g6**	478335_mir	hsa-miR-496
**B1h3**	477860_mir	hsa-miR-16-5p
**B2a2**	478326_mir	hsa-miR-370-3p
**B2a3**	478028_mir	hsa-miR-328-3p

**B2a4**	477959_mir	hsa-miR-197-3p
**B2a5**	478037_mir	hsa-miR-338-3p
**B2a6**	478043_mir	hsa-miR-342-3p
**B2a7**	478314_mir	hsa-miR-193b-3p
**B2a8**	478486_mir	hsa-miR-199b-5p
**B2b1**	477804_mir	hsa-miR-20b-5p
**B2b2**	478032_mir	hsa-miR-331-5p
**B2b3**	478293_mir	cel-miR-39-3p
**B2b4**	478479_mir	hsa-miR-33b-5p
**B2b5**	478067_mir	hsa-miR-369-3p
**B2b6**	478068_mir	hsa-miR-369-5p
**B2b7**	477819_mir	hsa-miR-208a-3p
**B2b8**	478337_mir	hsa-miR-542-5p
**B2c1**	478962_mir	hsa-miR-508-5p
**B2c2**	477906_mir	hsa-miR-139-3p
**B2c3**	479485_mir	hsa-miR-517a-3p_hsa-miR-517b-3p
**B2c4**	478989_mir	hsa-miR-520a-3p
**B2c5**	478231_mir	hsa-miR-199a-5p
**B2c6**	478997_mir	hsa-miR-526b-5p
**B2c7**	478585_mir	hsa-miR-196b-5p
**B2c8**	479340_mir	hsa-miR-519e-3p
**B2d1**	478995_mir	hsa-miR-525-3p
**B2d2**	478411_mir	ath-miR159a
**B2d3**	478984_mir	hsa-miR-518f-3p
**B2d4**	478153_mir	hsa-miR-542-3p
**B2d5**	479393_mir	hsa-miR-518d-3p
**B2d6**	478112_mir	hsa-miR-455-3p
**B2d7**	478685_mir	hsa-miR-1283
**B2d8**	478165_mir	hsa-miR-576-5p
**B2e1**	478351_mir	hsa-miR-200c-3p
**B2e2**	479396_mir	hsa-miR-525-5p
**B2e3**	479285_mir	hsa-miR-524-5p
**B2e4**	478210_mir	hsa-miR-93-5p
**B2e5**	478991_mir	hsa-miR-520g-3p
**B2e6**	479408_mir	hsa-miR-518e-3p
**B2e7**	479343_mir	hsa-miR-520f-3p
**B2e8**	477825_mir	hsa-miR-301b-3p
**B2f1**	478498_mir	hsa-miR-520e
**B2f2**	478914_mir	hsa-miR-450b-5p
**B2f3**	479053_mir	hsa-miR-570-3p
**B2f4**	478672_mir	hsa-miR-1267
**B2f5**	478156_mir	hsa-miR-544a
**B2f6**	479501_mir	hsa-miR-548a-5p
**B2f7**	477833_mir	hsa-miR-548d-3p
**B2f8**	478418_mir	hsa-miR-26b-5p
**B2g3**	478544_mir	hsa-miR-129-2-3p
**B2g4**	477835_mir	hsa-miR-487b-3p
**B2g5**	478132_mir	hsa-miR-491-5p
**B2g6**	478139_mir	hsa-miR-499a-5p
**B2h3**	477860_mir	hsa-miR-16-5p
**B3a2**	478744_mir	hsa-miR-195-3p

**B3a3**	478049_mir	hsa-miR-34b-3p
**B3a4**	478035_mir	hsa-miR-337-3p
**B3a5**	479057_mir	hsa-miR-577
**B3a6**	478831_mir	hsa-miR-33a-3p
**B3a7**	478188_mir	hsa-miR-645
**B3a8**	478840_mir	hsa-miR-363-5p
**B3b1**	478317_mir	hsa-miR-20a-3p
**B3b2**	478750_mir	hsa-miR-19a-5p
**B3b3**	478293_mir	cel-miR-39-3p
**B3b4**	478710_mir	hsa-miR-135b-3p
**B3b5**	477915_mir	hsa-miR-145-3p
**B3b6**	477895_mir	hsa-miR-1290
**B3b7**	478055_mir	hsa-miR-361-3p
**B3b8**	479451_mir	hsa-miR-1296-5p
**B3c1**	477951_mir	hsa-miR-191-3p
**B3c2**	478725_mir	hsa-miR-154-3p
**B3c3**	478505_mir	hsa-miR-151a-5p
**B3c4**	478051_mir	hsa-miR-34c-3p
**B3c5**	477919_mir	hsa-miR-151a-3p
**B3c6**	478671_mir	hsa-miR-1265
**B3c7**	479478_mir	hsa-miR-181a-2-3p
**B3c8**	478729_mir	hsa-miR-182-3p
**B3d1**	479181_mir	hsa-miR-802
**B3d2**	478411_mir	ath-miR159a
**B3d3**	479205_mir	hsa-miR-92a-1-5p
**B3d4**	479175_mir	hsa-miR-767-3p
**B3d5**	478681_mir	hsa-miR-1278
**B3d6**	479191_mir	hsa-miR-888-3p
**B3d7**	478650_mir	hsa-miR-1243
**B3d8**	478209_mir	hsa-miR-93-3p
**B3e1**	479217_mir	hsa-miR-941
**B3e2**	479071_mir	hsa-miR-588
**B3e3**	479349_mir	hsa-miR-513c-5p
**B3e4**	479084_mir	hsa-miR-603
**B3e5**	479212_mir	hsa-miR-937-3p
**B3e6**	479100_mir	hsa-miR-617
**B3e7**	479117_mir	hsa-miR-635
**B3e8**	479098_mir	hsa-miR-614
**B3f1**	479113_mir	hsa-miR-630
**B3f2**	479069_mir	hsa-miR-586
**B3f3**	478168_mir	hsa-miR-590-3p
**B3f4**	479085_mir	hsa-miR-604
**B3f5**	477868_mir	hsa-miR-10b-3p
**B3f6**	478980_mir	hsa-miR-517-5p
**B3f7**	479075_mir	hsa-miR-592
**B3f8**	479097_mir	hsa-miR-613
**B3g3**	479043_mir	hsa-miR-557
**B3g4**	479025_mir	hsa-miR-548p
**B3g5**	478173_mir	hsa-miR-601
**B3g6**	479038_mir	hsa-miR-553
**B3h3**	477860_mir	hsa-miR-16-5p

**B4a2**	478727_mir	hsa-miR-16-1-3p
**B4a3**	478711_mir	hsa-miR-138-2-3p
**B4a4**	478719_mir	hsa-miR-148b-5p
**B4a5**	479051_mir	hsa-miR-569
**B4a6**	477914_mir	hsa-miR-144-5p
**B4b1**	478758_mir	hsa-miR-203b-5p
**B4b2**	478680_mir	hsa-miR-1276
**B4b3**	478293_mir	cel-miR-39-3p
**B4b4**	477943_mir	hsa-miR-188-5p
**B4b5**	478699_mir	hsa-miR-1304-5p
**B4b6**	477811_mir	hsa-miR-151b
**B4b7**	478147_mir	hsa-miR-515-5p
**B4c1**	477890_mir	hsa-miR-1275
**B4c2**	477936_mir	hsa-miR-183-3p
**B4c3**	478673_mir	hsa-miR-1270
**B4c4**	478647_mir	hsa-miR-1236-3p
**B4c5**	477933_mir	hsa-miR-181c-3p
**B4c6**	479078_mir	hsa-miR-595
**B4c7**	477944_mir	hsa-miR-18a-3p
**B4d1**	479091_mir	hsa-miR-609
**B4d2**	478411_mir	ath-miR159a
**B4d3**	479104_mir	hsa-miR-620
**B4d4**	479067_mir	hsa-miR-585-3p
**B4d5**	478691_mir	hsa-miR-1292-5p
**B4d6**	478362_mir	hsa-miR-548e-3p
**B4d7**	479024_mir	hsa-miR-548n
**B4e1**	479512_mir	hsa-miR-623
**B4e2**	479532_mir	hsa-miR-518f-5p
**B4e3**	478349_mir	hsa-miR-378a-3p
**B4e4**	479338_mir	hsa-miR-524-3p
**B4e5**	479110_mir	hsa-miR-626
**B4e6**	479020_mir	hsa-miR-548g-3p
**B4e7**	479297_mir	hsa-miR-513b-5p
**B4f1**	479082_mir	hsa-miR-600
**B4f2**	480874_mir	hsa-miR-548i
**B4f3**	479391_mir	hsa-miR-548l
**B4f4**	477852_mir	hsa-miR-550a-5p
**B4f5**	478990_mir	hsa-miR-520d-3p
**B4f6**	478951_mir	hsa-miR-500a-3p
**B4f7**	478988_mir	hsa-miR-519e-5p
**B4g3**	479054_mir	hsa-miR-571
**B4g4**	477848_mir	hsa-let-7d-3p
**B4g5**	479491_mir	hsa-miR-518e-5p_hsa-miR-519a-5p_hsa-miR-519b-5p_hsa-miR-519c-5p_hsa-miR-522-5p_hsa-miR-523-5p
**B4g6**	478198_mir	hsa-miR-7-1-3p
**B4h3**	477860_mir	hsa-miR-16-5p
**C1a2**	477887_mir	hsa-miR-126-3p
**C1a3**	478102_mir	hsa-miR-433-3p
**C1a4**	478109_mir	hsa-miR-452-5p
**C1a5**	478094_mir	hsa-miR-425-5p
**C1a6**	478006_mir	hsa-miR-302a-3p

**C1a7**	478074_mir	hsa-miR-375
**C1a8**	477853_mir	hsa-miR-323a-3p
**C1b1**	478105_mir	hsa-miR-448
**C1b2**	478240_mir	hsa-miR-376a-3p
**C1b3**	478293_mir	cel-miR-39-3p
**C1b4**	478889_mir	hsa-miR-431-5p
**C1b5**	478369_mir	hsa-miR-29b-3p
**C1b6**	478329_mir	hsa-miR-454-3p
**C1b7**	478015_mir	hsa-miR-31-5p
**C1b8**	478128_mir	hsa-miR-486-5p
**C1c1**	479483_mir	hsa-miR-513a-5p
**C1c2**	478078_mir	hsa-miR-382-5p
**C1c3**	479528_mir	hsa-miR-449b-5p
**C1c4**	478501_mir	hsa-miR-141-3p
**C1c5**	478026_mir	hsa-miR-32-5p
**C1c6**	478430_mir	hsa-miR-298
**C1c7**	478422_mir	hsa-miR-486-3p
**C1c8**	479229_mir	hsa-miR-29c-3p
**C1d1**	478581_mir	hsa-miR-135a-5p
**C1d2**	478411_mir	ath-miR159a
**C1d3**	478715_mir	hsa-miR-146b-3p
**C1d4**	478717_mir	hsa-miR-147b
**C1d5**	477892_mir	hsa-miR-128-3p
**C1d6**	477851_mir	hsa-miR-130a-3p
**C1d7**	478779_mir	hsa-miR-222-5p
**C1d8**	477912_mir	hsa-miR-143-3p
**C1e1**	477910_mir	hsa-miR-142-3p
**C1e2**	477905_mir	hsa-miR-138-5p
**C1e3**	478670_mir	hsa-miR-1264
**C1e4**	478589_mir	hsa-miR-548b-5p
**C1e5**	478514_mir	hsa-miR-147a
**C1e6**	478582_mir	hsa-miR-135b-5p
**C1e7**	477937_mir	hsa-miR-183-5p
**C1e8**	477942_mir	hsa-miR-188-3p
**C1f1**	478511_mir	hsa-miR-133a-3p
**C1f2**	477921_mir	hsa-miR-152-3p
**C1f3**	478159_mir	hsa-miR-551b-3p
**C1f4**	478721_mir	hsa-miR-150-3p
**C1f5**	477939_mir	hsa-miR-185-5p
**C1f6**	477952_mir	hsa-miR-191-5p
**C1f7**	480871_mir	hsa-miR-133b
**C1f8**	478358_mir	hsa-miR-190a-5p
**C1g3**	478575_mir	hsa-let-7a-5p
**C1g4**	478507_mir	hsa-miR-211-5p
**C1g5**	479059_mir	hsa-miR-579-3p
**C1g6**	479042_mir	hsa-miR-556-5p
**C1h3**	477860_mir	hsa-miR-16-5p
**C2a2**	478085_mir	hsa-miR-410-3p
**C2a3**	477826_mir	hsa-miR-487a-3p
**C2a4**	477999_mir	hsa-miR-28-3p
**C2a5**	478079_mir	hsa-miR-383-5p

**C2a6**	478254_mir	hsa-miR-107
**C2a7**	478080_mir	hsa-miR-384
**C2a8**	478913_mir	hsa-miR-450b-3p
**C2b1**	477995_mir	hsa-miR-26a-5p
**C2b2**	478327_mir	hsa-miR-423-3p
**C2b3**	478293_mir	cel-miR-39-3p
**C2b4**	479241_mir	hsa-miR-10a-5p
**C2b5**	478130_mir	hsa-miR-489-3p
**C2b6**	477982_mir	hsa-miR-222-3p
**C2b7**	477865_mir	hsa-miR-105-5p
**C2b8**	477981_mir	hsa-miR-221-3p
**C2c1**	477884_mir	hsa-miR-125a-5p
**C2c2**	478494_mir	hsa-miR-10b-5p
**C2c3**	477815_mir	hsa-miR-301a-3p
**C2c4**	477934_mir	hsa-miR-181c-5p
**C2c5**	478942_mir	hsa-miR-491-3p
**C2c6**	477979_mir	hsa-miR-219a-2-3p
**C2c7**	478516_mir	hsa-miR-215-5p
**C2c8**	478270_mir	hsa-miR-27b-3p
**C2d1**	478368_mir	hsa-miR-654-5p
**C2d2**	478411_mir	ath-miR159a
**C2d3**	478412_mir	hsa-miR-106b-5p
**C2d4**	477864_mir	hsa-miR-103a-2-5p
**C2d5**	477885_mir	hsa-miR-125b-5p
**C2d6**	478192_mir	hsa-miR-660-5p
**C2d7**	478038_mir	hsa-miR-338-5p
**C2d8**	478197_mir	hsa-miR-708-5p
**C2e1**	478194_mir	hsa-miR-671-3p
**C2e2**	477922_mir	hsa-miR-153-3p
**C2e3**	477975_mir	hsa-miR-21-5p
**C2e4**	479135_mir	hsa-miR-654-3p
**C2e5**	478048_mir	hsa-miR-34a-5p
**C2e6**	479192_mir	hsa-miR-888-5p
**C2e7**	478189_mir	hsa-miR-652-3p
**C2e8**	477823_mir	hsa-miR-92b-3p
**C2f1**	479188_mir	hsa-miR-885-3p
**C2f2**	478204_mir	hsa-miR-873-5p
**C2f3**	477855_mir	hsa-miR-122-5p
**C2f4**	479123_mir	hsa-miR-643
**C2f5**	479187_mir	hsa-miR-876-5p
**C2f6**	478583_mir	hsa-miR-181b-5p
**C2f7**	478208_mir	hsa-miR-889-3p
**C2f8**	477827_mir	hsa-miR-92a-3p
**C2g3**	479166_mir	hsa-miR-758-3p
**C2g4**	479194_mir	hsa-miR-890
**C2g5**	477967_mir	hsa-miR-205-5p
**C2g6**	477941_mir	hsa-miR-187-3p
**C2h3**	477860_mir	hsa-miR-16-5p
**C3a2**	479421_mir	hsa-miR-374b-3p
**C3a3**	478863_mir	hsa-miR-377-5p
**C3a4**	478917_mir	hsa-miR-452-3p

**C3a5**	479049_mir	hsa-miR-567
**C3a6**	478798_mir	hsa-miR-302a-5p
**C3a7**	478804_mir	hsa-miR-30b-3p
**C3a8**	478093_mir	hsa-miR-425-3p
**C3b1**	479178_mir	hsa-miR-770-5p
**C3b2**	478003_mir	hsa-miR-29b-2-5p
**C3b3**	478293_mir	cel-miR-39-3p
**C3b4**	479412_mir	hsa-miR-30c-1-3p
**C3b5**	478892_mir	hsa-miR-432-3p
**C3b6**	478827_mir	hsa-miR-32-3p
**C3b7**	478919_mir	hsa-miR-454-5p
**C3b8**	478606_mir	hsa-miR-30d-5p
**C3c1**	478865_mir	hsa-miR-380-5p
**C3c2**	478786_mir	hsa-miR-25-5p
**C3c3**	478588_mir	hsa-miR-320b
**C3c4**	478623_mir	hsa-miR-106a-3p
**C3c5**	478794_mir	hsa-miR-29b-1-5p
**C3c6**	478768_mir	hsa-miR-214-5p
**C3c7**	478629_mir	hsa-miR-1184
**C3c8**	477801_mir	hsa-let-7f-1-3p
**C3d1**	478122_mir	hsa-miR-483-3p
**C3d2**	478411_mir	ath-miR159a
**C3d3**	478734_mir	hsa-miR-18b-3p
**C3d4**	478665_mir	hsa-miR-125b-1-3p
**C3d5**	478653_mir	hsa-miR-1248
**C3d6**	477926_mir	hsa-miR-155-3p
**C3d7**	479185_mir	hsa-miR-875-5p
**C3d8**	478658_mir	hsa-miR-1252-5p
**C3e1**	478660_mir	hsa-miR-1254
**C3e2**	478662_mir	hsa-miR-1255b-5p
**C3e3**	477929_mir	hsa-miR-15b-3p
**C3e4**	478641_mir	hsa-miR-1226-5p
**C3e5**	477880_mir	hsa-miR-124-5p
**C3e6**	477875_mir	hsa-miR-1225-3p
**C3e7**	478644_mir	hsa-miR-1228-5p
**C3e8**	478657_mir	hsa-miR-1251-5p
**C3f1**	477874_mir	hsa-miR-122-3p
**C3f2**	478730_mir	hsa-miR-1825
**C3f3**	479120_mir	hsa-miR-641
**C3f4**	479176_mir	hsa-miR-767-5p
**C3f5**	478661_mir	hsa-miR-1255a
**C3f6**	479128_mir	hsa-miR-649
**C3f7**	478638_mir	hsa-miR-1224-3p
**C3f8**	478340_mir	hsa-miR-631
**C3g3**	478637_mir	hsa-miR-1208
**C3g4**	478161_mir	hsa-miR-564
**C3g5**	479063_mir	hsa-miR-581
**C3g6**	479040_mir	hsa-miR-555
**C3h3**	477860_mir	hsa-miR-16-5p
**C4a2**	477973_mir	hsa-miR-21-3p
**C4a3**	478631_mir	hsa-miR-1200

**C4a4**	478788_mir	hsa-miR-26a-2-3p
**C4a5**	479058_mir	hsa-miR-578
**C4a6**	477862_mir	hsa-let-7i-3p
**C4a7**	477850_mir	hsa-let-7g-3p
**C4b1**	477869_mir	hsa-miR-1180-3p
**C4b2**	478341_mir	hsa-miR-7-5p
**C4b3**	478293_mir	cel-miR-39-3p
**C4b4**	478221_mir	hsa-let-7b-3p
**C4b5**	478787_mir	hsa-miR-26a-1-3p
**C4b6**	477984_mir	hsa-miR-223-5p
**C4b7**	478784_mir	hsa-miR-24-1-5p
**C4c1**	478630_mir	hsa-miR-1197
**C4c2**	478755_mir	hsa-miR-202-5p
**C4c3**	478774_mir	hsa-miR-218-1-3p
**C4c4**	478745_mir	hsa-miR-196a-3p
**C4c5**	478625_mir	hsa-miR-1178-3p
**C4c6**	478713_mir	hsa-miR-143-5p
**C4c7**	478846_mir	hsa-miR-367-5p
**C4d1**	479115_mir	hsa-miR-633
**C4d2**	478411_mir	ath-miR159a
**C4d3**	479125_mir	hsa-miR-646
**C4d4**	479150_mir	hsa-miR-665
**C4d5**	479139_mir	hsa-miR-657
**C4d6**	479116_mir	hsa-miR-634
**C4d7**	477899_mir	hsa-miR-130b-5p
**C4e1**	479140_mir	hsa-miR-658
**C4e2**	479127_mir	hsa-miR-648
**C4e3**	479151_mir	hsa-miR-668-3p
**C4e4**	479146_mir	hsa-miR-663b
**C4e5**	477958_mir	hsa-miR-196b-3p
**C4e6**	478342_mir	hsa-miR-766-3p
**C4e7**	479119_mir	hsa-miR-640
**C4f1**	479204_mir	hsa-miR-924
**C4f2**	479206_mir	hsa-miR-92a-2-5p
**C4f3**	479210_mir	hsa-miR-935
**C4f4**	479452_mir	hsa-miR-1298-5p
**C4f5**	479220_mir	hsa-miR-944
**C4f6**	479214_mir	hsa-miR-938
**C4f7**	479198_mir	hsa-miR-892b
**C4g3**	479048_mir	hsa-miR-563
**C4g4**	479065_mir	hsa-miR-583
**C4g5**	479055_mir	hsa-miR-573
**C4g6**	479039_mir	hsa-miR-554
**C4h3**	477860_mir	hsa-miR-16-5p
**D1a2**	477836_mir	hsa-miR-296-5p
**D1a3**	477820_mir	hsa-miR-1-3p
**D1a4**	479456_mir	hsa-miR-216b-5p
**D1a5**	477983_mir	hsa-miR-223-3p
**D1a6**	478029_mir	hsa-miR-329-3p
**D1a7**	477992_mir	hsa-miR-24-3p
**D1a8**	477863_mir	hsa-miR-101-3p

**D1b1**	477977_mir	hsa-miR-218-5p
**D1b2**	477994_mir	hsa-miR-25-3p
**D1b3**	478293_mir	cel-miR-39-3p
**D1b4**	478225_mir	hsa-miR-106a-5p
**D1b5**	478384_mir	hsa-miR-27a-3p
**D1b6**	477961_mir	hsa-miR-199a-3p_hsa-miR-199b-3p
**D1b7**	479544_mir	hsa-miR-186-3p
**D1b8**	478363_mir	hsa-miR-373-3p
**D1c1**	478490_mir	hsa-miR-200a-3p
**D1c2**	478238_mir	hsa-miR-374a-5p
**D1c3**	478070_mir	hsa-miR-371a-3p
**D1c4**	478025_mir	hsa-miR-325
**D1c5**	478059_mir	hsa-miR-362-5p
**D1c6**	478979_mir	hsa-miR-516b-5p
**D1c7**	478264_mir	hsa-miR-19b-3p
**D1c8**	478323_mir	hsa-miR-331-3p
**D1d1**	478134_mir	hsa-miR-493-3p
**D1d2**	478411_mir	ath-miR159a
**D1d3**	478350_mir	hsa-miR-501-3p
**D1d4**	478972_mir	hsa-miR-512-5p
**D1d5**	478960_mir	hsa-miR-507
**D1d6**	478145_mir	hsa-miR-505-3p
**D1d7**	479089_mir	hsa-miR-608
**D1d8**	478982_mir	hsa-miR-518c-3p
**D1e1**	478142_mir	hsa-miR-501-5p
**D1e2**	479530_mir	hsa-miR-518d-5p_hsa-miR-520c-5p_hsa-miR-526a
**D1e3**	478958_mir	hsa-miR-506-3p
**D1e4**	478309_mir	hsa-miR-500a-5p
**D1e5**	478971_mir	hsa-miR-512-3p
**D1e6**	479101_mir	hsa-miR-618
**D1e7**	478088_mir	hsa-miR-421
**D1e8**	478968_mir	hsa-miR-510-5p
**D1f1**	478367_mir	hsa-miR-590-5p
**D1f2**	479073_mir	hsa-miR-589-5p
**D1f3**	479469_mir	hsa-miR-625-5p
**D1f4**	478977_mir	hsa-miR-516a-3p_hsa-miR-516b-3p
**D1f5**	478177_mir	hsa-miR-616-3p
**D1f6**	478215_mir	hsa-miR-96-5p
**D1f7**	478427_mir	hsa-miR-627-5p
**D1f8**	478348_mir	hsa-miR-502-3p
**D1g3**	479064_mir	hsa-miR-582-3p
**D1g4**	478077_mir	hsa-miR-379-5p
**D1g5**	477879_mir	hsa-miR-124-3p
**D1g6**	478532_mir	hsa-miR-23a-3p
**D1h3**	477860_mir	hsa-miR-16-5p
**D2a2**	478586_mir	hsa-miR-20a-5p
**D2a3**	477904_mir	hsa-miR-137
**D2a4**	478136_mir	hsa-miR-495-3p
**D2a5**	478060_mir	hsa-miR-363-3p
**D2a6**	477814_mir	hsa-miR-148a-3p
**D2a7**	478030_mir	hsa-miR-330-3p

**D2a8**	478066_mir	hsa-miR-367-3p
**D2b1**	478324_mir	hsa-miR-335-5p
**D2b2**	478325_mir	hsa-miR-339-3p
**D2b3**	478293_mir	cel-miR-39-3p
**D2b4**	478052_mir	hsa-miR-34c-5p
**D2b5**	477957_mir	hsa-miR-195-5p
**D2b6**	478071_mir	hsa-miR-372-3p
**D2b7**	477917_mir	hsa-miR-149-5p
**D2b8**	477954_mir	hsa-miR-193a-5p
**D2c1**	478307_mir	hsa-miR-136-5p
**D2c2**	478058_mir	hsa-miR-362-3p
**D2c3**	478040_mir	hsa-miR-339-5p
**D2c4**	478830_mir	hsa-miR-330-5p
**D2c5**	478986_mir	hsa-miR-519d-3p
**D2c6**	479228_mir	hsa-miR-19a-3p
**D2c7**	478306_mir	hsa-miR-193a-3p
**D2c8**	477956_mir	hsa-miR-194-5p
**D2d1**	478970_mir	hsa-miR-511-5p
**D2d2**	478411_mir	ath-miR159a
**D2d3**	479249_mir	hsa-miR-518a-5p_hsa-miR-527
**D2d4**	478616_mir	hsa-miR-520d-5p
**D2d5**	477927_mir	hsa-miR-155-5p
**D2d6**	478999_mir	hsa-miR-541-3p
**D2d7**	479003_mir	hsa-miR-545-5p
**D2d8**	478994_mir	hsa-miR-523-3p
**D2e1**	478152_mir	hsa-miR-539-5p
**D2e2**	478336_mir	hsa-miR-532-3p
**D2e3**	479495_mir	hsa-miR-519c-3p
**D2e4**	479002_mir	hsa-miR-545-3p
**D2e5**	479509_mir	hsa-miR-520b
**D2e6**	479534_mir	hsa-miR-519a-3p
**D2e7**	478993_mir	hsa-miR-522-3p
**D2e8**	479404_mir	hsa-miR-520a-5p
**D2f1**	478149_mir	hsa-miR-521
**D2f2**	478163_mir	hsa-miR-574-3p
**D2f3**	478151_mir	hsa-miR-532-5p
**D2f4**	479503_mir	hsa-miR-548h-5p
**D2f5**	479018_mir	hsa-miR-548b-3p
**D2f6**	480872_mir	hsa-miR-548am-5p_hsa-miR-548c-5p_hsa-miR-548o-5p
**D2f7**	479041_mir	hsa-miR-556-3p
**D2f8**	478157_mir	hsa-miR-548a-3p
**D2g3**	478107_mir	hsa-miR-451a
**D2g4**	478214_mir	hsa-miR-9-5p
**D2g5**	478175_mir	hsa-miR-615-3p
**D2g6**	477901_mir	hsa-miR-134-5p
**D2h3**	477860_mir	hsa-miR-16-5p
**D3a2**	478752_mir	hsa-miR-200a-5p
**D3a3**	478753_mir	hsa-miR-200b-5p
**D3a4**	479032_mir	hsa-miR-550a-3p
**D3a5**	478741_mir	hsa-miR-192-3p
**D3a6**	478754_mir	hsa-miR-200c-5p

**D3a7**	478764_mir	hsa-miR-20b-3p
**D3a8**	478193_mir	hsa-miR-664a-3p
**D3b1**	477902_mir	hsa-miR-136-3p
**D3b2**	478712_mir	hsa-miR-141-5p
**D3b3**	478293_mir	cel-miR-39-3p
**D3b4**	478743_mir	hsa-miR-194-3p
**D3b5**	477968_mir	hsa-miR-206
**D3b6**	479036_mir	hsa-miR-552-3p
**D3b7**	478041_mir	hsa-miR-340-3p
**D3b8**	478347_mir	hsa-miR-33a-5p
**D3c1**	477913_mir	hsa-miR-144-3p
**D3c2**	478720_mir	hsa-miR-149-3p
**D3c3**	478693_mir	hsa-miR-1294
**D3c4**	478718_mir	hsa-miR-148a-5p
**D3c5**	478212_mir	hsa-miR-942-5p
**D3c6**	478697_mir	hsa-miR-1302
**D3c7**	478690_mir	hsa-miR-1291
**D3c8**	477931_mir	hsa-miR-16-2-3p
**D3d1**	479202_mir	hsa-miR-921
**D3d2**	478411_mir	ath-miR159a
**D3d3**	479208_mir	hsa-miR-933
**D3d4**	478686_mir	hsa-miR-1284
**D3d5**	479526_mir	hsa-miR-411-3p
**D3d6**	479201_mir	hsa-miR-920
**D3d7**	479173_mir	hsa-miR-765
**D3d8**	479211_mir	hsa-miR-936
**D3e1**	478946_mir	hsa-miR-497-3p
**D3e2**	479076_mir	hsa-miR-593-3p
**D3e3**	478050_mir	hsa-miR-34b-5p
**D3e4**	479088_mir	hsa-miR-607
**D3e5**	479087_mir	hsa-miR-606
**D3e6**	479106_mir	hsa-miR-622
**D3e7**	478983_mir	hsa-miR-518c-5p
**D3e8**	478181_mir	hsa-miR-628-3p
**D3f1**	479070_mir	hsa-miR-587
**D3f2**	479072_mir	hsa-miR-589-3p
**D3f3**	479077_mir	hsa-miR-593-5p
**D3f4**	479177_mir	hsa-miR-769-3p
**D3f5**	477893_mir	hsa-miR-1286
**D3f6**	478338_mir	hsa-miR-596
**D3f7**	479105_mir	hsa-miR-621
**D3f8**	478174_mir	hsa-miR-605-5p
**D3g3**	479047_mir	hsa-miR-562
**D3g4**	478158_mir	hsa-miR-551a
**D3g5**	478162_mir	hsa-miR-572
**D3g6**	479373_mir	hsa-miR-566
**D3h3**	477860_mir	hsa-miR-16-5p
**D4a1**	478216_mir	hsa-miR-99b-3p
**D4a2**	477932_mir	hsa-miR-17-3p
**D4a3**	478688_mir	hsa-miR-1288-3p
**D4a4**	478674_mir	hsa-miR-1271-5p

**D4a5**	479522_mir	hsa-miR-1269a
**D4a6**	480873_mir	hsa-miR-129-1-3p
**D4a7**	477897_mir	hsa-miR-1301-3p
**D4b1**	478732_mir	hsa-miR-185-3p
**D4b2**	478675_mir	hsa-miR-1272
**D4b3**	478293_mir	cel-miR-39-3p
**D4b4**	478714_mir	hsa-miR-146a-3p
**D4b5**	478230_mir	hsa-miR-196a-5p
**D4b6**	478687_mir	hsa-miR-1285-3p
**D4b7**	478692_mir	hsa-miR-1293
**D4c1**	477928_mir	hsa-miR-15a-3p
**D4c2**	478689_mir	hsa-miR-1289
**D4c3**	478659_mir	hsa-miR-1253
**D4c4**	478698_mir	hsa-miR-1303
**D4c5**	478310_mir	hsa-miR-1203
**D4c6**	478683_mir	hsa-miR-1282
**D4c7**	478664_mir	hsa-miR-1257
**D4d1**	479099_mir	hsa-miR-616-5p
**D4d2**	478411_mir	ath-miR159a
**D4d3**	478178_mir	hsa-miR-624-5p
**D4d4**	479074_mir	hsa-miR-591
**D4d5**	478182_mir	hsa-miR-629-3p
**D4d6**	479081_mir	hsa-miR-599
**D4e1**	478179_mir	hsa-miR-625-3p
**D4e2**	479499_mir	hsa-miR-520h
**D4e3**	478138_mir	hsa-miR-497-5p
**D4e4**	478155_mir	hsa-miR-543
**D4e5**	478957_mir	hsa-miR-505-5p
**D4e6**	479022_mir	hsa-miR-548j-5p
**D4f1**	479023_mir	hsa-miR-548m
**D4f2**	479000_mir	hsa-miR-541-5p
**D4f3**	479536_mir	hsa-miR-520c-3p
**D4f4**	478705_mir	hsa-miR-132-5p
**D4f5**	479333_mir	hsa-miR-519b-3p
**D4f6**	479374_mir	hsa-miR-548k
**D4g3**	479056_mir	hsa-miR-575
**D4g4**	479222_mir	hsa-miR-96-3p
**D4g5**	479224_mir	hsa-miR-99a-3p
**D4g6**	478005_mir	hsa-miR-29c-5p
**D4h3**	477860_mir	hsa-miR-16-5p

Table S2. Taqman advanced miRNA assays and associated assay IDs used in the study. Acquired from ThermoFisher Scientific.

miRNA	Assay ID
hsa-miR-324-5p	483066_mir
hsa-miR-29b-3p	478794_mir
hsa-miR-19a-3p	479228_mir
hsa-miR-186-5p	477940_mir
hsa-miR-361-5p	478056_mir
hsa-miR-425-5p	478094_mir
hsa-miR-187-3p	477941_mir
hsa-miR-29a-3p	478587_mir
hsa-miR-660-5p	478192_mir
hsa-miR-155-5p	483064_mir
hsa-miR-181a-5p	477857_mir
hsa-miR-222-3p	477982_mir
hsa-miR-339-5p	478040_mir
hsa-miR-25-3p	477994_mir
hsa-miR-92a-3p	477827_mir
hsa-miR-92b-3p	477823_mir
hsa-miR-22-5p	477987_mir
hsa-miR-29c-5p	478005_mir
hsa-miR-125a-5p	477884_mir
ath-miR159a	478411_mir

Table S3. Raw CT values for 4 candidate miRNAs and endogenous control miRNA hsa-miR-125a-5p in the validation cohort. CT values in bold were missings replaced by maximum CT value + 1. Dense category (extremely high or very low), age, body mass index (BMI), Parity and NAF color category are provided. UD = undetermined.

	Dense category	Age	BMI	Parity	NAF colour	hsa-miR-92a-3p	hsa-miR-92b-3p	hsa-miR-22-5p	hsa-miR-29c-5p	hsa-miR-125a-5p
**sample 1**	HIGH	53	22.2	Parous	green/brown	20.3	21.3	27.8	32.3	19.6
**sample 2**	LOW	51	40.9	Nulliparous	green/brown	21.0	21.0	26.0	28.9	18.9
**sample 3**	LOW	52	26.9	Parous	cloudy white/yellow	25.1	25.3	**35.0**	31.0	28.0
**sample 4**	HIGH	50	20.5	Parous	green/brown	19.5	20.2	25.9	25.7	18.1
**sample 5**	HIGH	51	23.1	Parous	bloody/orange/pink	17.4	19.0	22.7	26.2	19.8
**sample 6**	HIGH	66	24.5	Parous	bloody/orange/pink	17.6	19.1	23.5	26.8	20.3
**sample 7**	HIGH	53	24.5	Parous	clear white/yellow	16.3	17.3	22.1	25.3	19.5
**sample 8**	HIGH	69	19.4	Parous	bloody/orange/pink	17.4	18.5	23.7	25.8	19.9
**sample 9**	HIGH	59	UD	UD	bloody/orange/pink	17.7	19.4	23.2	26.2	21.3
**sample 10**	HIGH	50	22.3	Parous	green/brown	18.5	19.7	20.0	33.0	21.9
**sample 11**	HIGH	52	22	Nulliparous	cloudy white/yellow	19.7	20.7	24.9	27.9	18.9
**sample 12**	HIGH	69	21.5	Parous	bloody/orange/pink	16.8	18.1	22.9	25.0	18.0
**sample 13**	HIGH	52	24.6	Parous	clear white/yellow	17.5	18.7	22.5	28.7	20.4
**sample 14**	LOW	53	35.9	Parous	green/brown	21.4	21.8	28.5	30.3	20.3
**sample 15**	LOW	50	27.5	Parous	cloudy white/yellow	19.0	20.0	25.4	27.6	19.0
**sample 16**	LOW	52	24.9	Parous	green/brown	20.4	21.3	25.5	29.4	19.4
**sample 17**	LOW	59	35.6	Parous	clear white/yellow	20.5	21.2	24.7	28.8	21.7
**sample 18**	LOW	54	28.6	Nulliparous	cloudy white/yellow	18.3	19.0	24.1	28.9	20.3
**sample 19**	LOW	60	30.5	Parous	bloody/orange/pink	17.6	18.6	20.7	25.3	19.4
**sample 20**	LOW	56	28.7	Parous	clear white/yellow	18.8	20.0	23.4	27.6	20.2
**sample 21**	LOW	55	25.9	Parous	clear white/yellow	18.8	19.9	23.6	27.9	20.4
**sample 22**	LOW	56	25	Parous	clear white/yellow	22.1	22.3	**35.0**	30.5	21.0
**sample 23**	HIGH	57	26.2	Parous	clear white/yellow	17.7	19.0	22.5	25.4	19.2
**sample 24**	HIGH	53	UD	UD	bloody/orange/pink	18.0	18.9	24.3	27.4	19.4
**sample 25**	HIGH	58	20.7	Parous	bloody/orange/pink	16.6	18.2	22.7	24.9	18.1
**sample 26**	LOW	50	29.8	Parous	clear white/yellow	17.2	18.4	21.6	25.5	19.2
**sample 27**	LOW	52	27	Parous	cloudy white/yellow	19.0	20.1	23.7	27.1	18.1
**sample 28**	LOW	58	UD	Parous	bloody/orange/pink	17.3	18.7	22.4	26.7	19.2
**sample 29**	HIGH	54	19.7	Parous	cloudy white/yellow	20.2	21.2	24.2	28.0	19.8
**sample 30**	HIGH	67	24	Parous	cloudy white/yellow	20.1	20.9	26.6	29.4	18.6
**sample 31**	HIGH	50	26.5	Parous	bloody/orange/pink	17.9	19.1	22.3	26.1	19.7
**sample 32**	LOW	56	30.7	Parous	cloudy white/yellow	21.7	22.5	26.5	31.5	21.4
**sample 33**	LOW	59	26.6	Parous	cloudy white/yellow	18.6	20.1	26.2	28.7	19.2
**sample 34**	LOW	57	28.4	Parous	clear white/yellow	20.8	21.1	23.9	27.6	22.0
**sample 35**	HIGH	74	20.8	Parous	clear white/yellow	17.4	18.4	23.7	26.2	19.1
**sample 36**	HIGH	54	20.8	Parous	green/brown	20.4	21.4	31.6	30.7	19.1
**sample 37**	HIGH	53	25.2	Parous	cloudy white/yellow	20.2	20.4	22.1	25.7	21.0
**sample 38**	HIGH	62	22.3	Parous	clear white/yellow	17.1	18.2	22.9	26.4	18.4
**sample 39**	LOW	55	28.7	Parous	cloudy white/yellow	22.9	23.1	27.9	32.2	21.2
**sample 40**	LOW	52	28.3	Parous	cloudy white/yellow	17.8	19.0	22.9	34.0	20.3
**sample 41**	LOW	50	24.7	Parous	clear white/yellow	17.6	18.8	21.8	27.1	19.4

**sample 42**	LOW	54	32.5	Parous	clear white/yellow	17.9	19.1	22.2	25.7	20.5
**sample 43**	HIGH	70	22	Parous	cloudy white/yellow	17.7	19.1	23.7	27.0	19.2
**sample 44**	HIGH	54	20.9	Parous	clear white/yellow	22.8	23.5	28.5	29.7	22.8
**sample 45**	LOW	55	38.5	Parous	bloody/orange/pink	17.9	18.8	23.8	29.8	21.4
**sample 46**	LOW	52	UD	Parous	cloudy white/yellow	23.1	23.8	28.5	34.3	23.6
**sample 47**	HIGH	70	24.8	Parous	bloody/orange/pink	17.1	19.1	22.4	25.6	19.6
**sample 48**	HIGH	62	23.6	Parous	bloody/orange/pink	17.6	19.3	23.1	25.7	20.1
**sample 49**	LOW	55	37.4	Parous	bloody/orange/pink	18.7	20.7	24.4	26.6	21.5
**sample 50**	LOW	50	26.8	Parous	bloody/orange/pink	17.2	19.1	21.8	24.7	20.6
**sample 51**	HIGH	63	18.8	Parous	clear white/yellow	17.9	20.2	24.4	26.0	20.0
**sample 52**	HIGH	55	21.6	Nulliparous	cloudy white/yellow	20.9	21.8	26.2	28.1	21.7
**sample 53**	LOW	53	25.3	Parous	bloody/orange/pink	19.0	21.0	24.8	29.3	22.3
**sample 54**	LOW	57	26.8	Parous	clear white/yellow	UD	UD	UD	UD	UD
**sample 55**	HIGH	64	25	Parous	bloody/orange/pink	17.2	19.1	22.2	24.3	19.7
**sample 56**	HIGH	57	24.2	Parous	cloudy white/yellow	20.8	22.0	26.3	28.6	20.8
**sample 57**	LOW	55	31.3	Parous	bloody/orange/pink	17.4	19.0	22.5	25.6	20.0
**sample 58**	LOW	52	24.7	Parous	cloudy white/yellow	18.0	20.1	23.8	26.6	21.1
**sample 59**	HIGH	51	19.6	Parous	green/brown	23.8	**28.0**	30.6	32.0	24.3
**sample 60**	LOW	60	49.6	Parous	cloudy white/yellow	23.0	23.4	31.4	29.1	23.1
**sample 61**	HIGH	52	21.1	Parous	clear white/yellow	18.3	19.9	24.5	28.5	20.8
**sample 62**	LOW	58	28.7	Parous	clear white/yellow	20.9	22.0	29.8	32.3	24.5
**sample 63**	LOW	52	UD	Parous	bloody/orange/pink	18.2	19.8	23.1	26.0	20.9
**sample 64**	LOW	52	34.3	Parous	clear white/yellow	18.0	19.2	24.4	26.6	20.3
**sample 65**	LOW	55	24.6	Parous	green/brown	19.8	20.9	26.7	33.1	21.5
**sample 66**	HIGH	50	23.6	Parous	clear white/yellow	21.2	22.3	26.7	29.9	24.4
**sample 67**	HIGH	62	21.4	Nulliparous	clear white/yellow	17.6	19.4	23.2	25.2	20.8
**sample 68**	HIGH	59	20.5	Parous	bloody/orange/pink	18.2	19.6	24.6	28.9	21.0
**sample 69**	HIGH	57	21.3	Parous	clear white/yellow	18.3	19.9	24.3	27.5	20.3
**sample 70**	HIGH	63	UD	UD	bloody/orange/pink	17.3	19.2	24.5	27.0	19.7
**sample 71**	LOW	57	28.3	Parous	cloudy white/yellow	25.5	26.1	31.9	33.3	25.3
**sample 72**	HIGH	53	21.8	Nulliparous	green/brown	22.6	22.7	28.2	31.3	22.4
**sample 73**	LOW	50	38.4	Nulliparous	bloody/orange/pink	18.8	19.9	25.0	26.8	21.5
**sample 74**	LOW	60	33.7	Parous	bloody/orange/pink	17.8	19.7	24.1	26.1	20.6
**sample 75**	HIGH	50	20.8	Parous	clear white/yellow	18.8	19.7	26.5	27.2	19.4
**sample 76**	HIGH	50	22.8	Parous	bloody/orange/pink	16.9	18.6	22.8	24.8	19.7
**sample 77**	LOW	59	UD	Parous	clear white/yellow	18.4	20.3	24.2	26.8	20.5
**sample 78**	LOW	58	25.4	Parous	bloody/orange/pink	17.6	19.5	23.2	24.8	19.5
**sample 79**	HIGH	51	22	Parous	cloudy white/yellow	22.4	23.1	28.3	27.7	21.9
**sample 80**	HIGH	53	21	Parous	cloudy white/yellow	19.0	20.2	24.4	25.5	18.2
**sample 81**	HIGH	56	34.5	Parous	clear white/yellow	20.8	21.7	25.2	27.4	20.5
**sample 82**	LOW	57	28.3	Parous	clear white/yellow	22.7	23.5	27.7	29.3	21.9
**sample 83**	LOW	60	29.4	Parous	clear white/yellow	24.6	24.7	31.3	32.6	25.0
**sample 84**	HIGH	59	21.8	Parous	bloody/orange/pink	17.3	19.3	23.6	26.3	21.7
**sample 85**	HIGH	54	20.7	Parous	clear white/yellow	17.3	19.1	**35.0**	25.5	20.3
**sample 86**	LOW	53	32.3	Parous	green/brown	UD	UD	UD	UD	UD
**sample 87**	LOW	55	30.5	Parous	cloudy white/yellow	21.2	22.1	29.4	**35.0**	23.0
**sample 88**	HIGH	51	23.7	Parous	clear white/yellow	UD	UD	UD	UD	UD
**sample 89**	HIGH	54	20.3	Parous	cloudy white/yellow	24.9	23.4	31.3	32.8	24.7
**sample 90**	LOW	57	31.4	Nulliparous	cloudy white/yellow	25.2	23.6	32.3	**35.0**	25.3
**sample 91**	LOW	55	40.8	Nulliparous	clear white/yellow	18.0	18.0	23.5	25.9	20.2
**sample 92**	HIGH	51	22	Nulliparous	cloudy white/yellow	20.0	20.3	25.4	25.8	22.0

**sample 93**	HIGH	73	18.3	Parous	cloudy white/yellow	22.8	21.9	**35.0**	29.7	21.7
**sample 94**	LOW	57	35.4	Parous	clear white/yellow	22.1	21.4	29.1	**35.0**	23.8
**sample 95**	LOW	54	48.1	Parous	cloudy white/yellow	22.8	22.4	**35.0**	30.1	22.1
**sample 96**	HIGH	56	20.7	Parous	bloody/orange/pink	17.1	17.4	26.2	24.8	18.7
**sample 97**	HIGH	52	25.3	Parous	cloudy white/yellow	18.4	18.8	24.4	26.6	20.0
**sample 98**	LOW	60	27	Parous	clear white/yellow	UD	UD	UD	UD	UD
**sample 99**	LOW	54	27.2	Parous	cloudy white/yellow	22.9	22.2	29.1	**35.0**	24.0
**sample 100**	HIGH	57	21	Parous	clear white/yellow	17.3	17.7	22.7	25.5	18.7
**sample 101**	HIGH	54	24.1	Parous	clear white/yellow	18.5	18.5	24.5	26.8	20.3
**sample 102**	LOW	53	32	Parous	clear white/yellow	18.1	18.5	23.6	26.8	21.3
**sample 103**	LOW	57	28	Parous	clear white/yellow	18.7	18.8	23.6	27.2	21.6
**sample 104**	HIGH	61	17	Parous	clear white/yellow	17.5	17.6	25.0	25.0	20.5
**sample 105**	HIGH	52	UD	UD	cloudy white/yellow	19.3	19.5	24.3	24.7	22.5
**sample 106**	LOW	53	35.2	Parous	clear white/yellow	17.5	17.8	23.4	24.9	19.1
**sample 107**	LOW	56	30.4	Parous	clear white/yellow	19.2	19.4	26.0	28.6	22.1
**sample 108**	HIGH	51	24.8	Nulliparous	clear white/yellow	30.8	UD	UD	UD	30.9
**sample 109**	HIGH	57	19.4	Parous	clear white/yellow	25.0	23.6	**35.0**	33.0	23.4
**sample 110**	LOW	53	30.3	Parous	clear white/yellow	18.2	18.6	23.0	25.3	21.5
**sample 111**	LOW	55	UD	UD	clear white/yellow	18.9	19.2	24.2	26.8	21.5
**sample 112**	HIGH	50	19.1	Parous	clear white/yellow	20.4	20.1	26.3	28.4	25.0
**sample 113**	HIGH	57	20.4	Parous	cloudy white/yellow	26.7	25.5	31.9	32.9	27.6
**sample 114**	LOW	59	23.6	Parous	clear white/yellow	19.5	19.3	25.6	27.8	21.9
**sample 115**	LOW	54	24.6	Parous	bloody/orange/pink	18.9	19.3	23.0	25.9	22.2
**sample 116**	HIGH	54	23.1	Parous	clear white/yellow	18.2	18.7	24.3	25.5	20.3
**sample 117**	LOW	52	29	Parous	cloudy white/yellow	20.9	20.9	27.9	28.7	25.4
**sample 118**	LOW	59	UD	Parous	clear white/yellow	32.5	UD	UD	UD	UD
**sample 119**	HIGH	59	23.7	Nulliparous	cloudy white/yellow	23.9	23.1	30.0	29.0	24.4
**sample 120**	HIGH	50	24.3	Parous	green/brown	24.2	23.5	31.8	33.1	24.1
**sample 121**	LOW	50	46.1	Parous	clear white/yellow	22.2	22.0	29.1	29.4	23.1
**sample 122**	LOW	50	35.1	Parous	cloudy white/yellow	23.2	22.6	**35.0**	31.2	23.6
**sample 123**	HIGH	55	UD	UD	bloody/orange/pink	17.5	18.2	27.4	26.4	20.5
**sample 124**	HIGH	63	21.3	Parous	cloudy white/yellow	19.8	19.9	24.0	24.8	22.8
**sample 125**	LOW	57	28.4	Parous	cloudy white/yellow	23.5	22.9	30.1	30.8	26.4
**sample 126**	LOW	50	26	Nulliparous	cloudy white/yellow	24.7	23.9	29.5	29.9	26.4
**sample 127**	HIGH	52	25.6	Parous	clear white/yellow	21.5	21.5	28.5	30.7	23.9
**sample 128**	HIGH	57	25.1	Parous	blue > not categorized	24.4	23.7	33.2	33.0	27.7
**sample 129**	LOW	53	29.1	Parous	clear white/yellow	23.5	23.4	33.8	**35.0**	25.4
**sample 130**	LOW	59	24.4	Nulliparous	cloudy white/yellow	22.2	22.2	28.9	30.5	23.4
**sample 131**	HIGH	66	24	Nulliparous	clear white/yellow	18.9	20.4	27.1	30.3	22.9
**sample 132**	HIGH	51	17.8	Nulliparous	clear white/yellow	UD	UD	UD	UD	UD
**sample 133**	LOW	56	43.3	Parous	clear white/yellow	21.1	**28.0**	24.0	27.2	24.5
**sample 134**	LOW	50	23.5	Parous	cloudy white/yellow	23.6	23.5	29.4	33.9	23.7
**sample 135**	HIGH	58	20.7	Nulliparous	clear white/yellow	24.3	23.8	31.1	31.9	25.9
**sample 136**	HIGH	52	21.2	Parous	clear white/yellow	21.2	21.8	26.8	30.5	25.0
**sample 137**	LOW	57	29.1	Nulliparous	clear white/yellow	20.0	21.1	25.7	27.8	23.7
**sample 138**	HIGH	61	19.5	Parous	clear white/yellow	24.8	24.4	30.9	31.1	25.5
**sample 139**	HIGH	64	21.6	Parous	clear white/yellow	24.1	24.1	30.0	33.1	29.0
**sample 140**	LOW	55		Parous	clear white/yellow	UD	UD	UD	UD	UD
**sample 141**	LOW	54		Parous	clear white/yellow	UD	UD	UD	UD	UD
**sample 142**	HIGH	54	25.4	Nulliparous	cloudy white/yellow	19.0	20.0	24.5	27.1	22.2
**sample 143**	HIGH	61	21.1	Nulliparous	cloudy white/yellow	22.4	22.5	29.6	29.6	22.5

**sample 144**	LOW	52	29.8	Parous	clear white/yellow	18.9	20.1	24.8	27.5	22.0
**sample 145**	HIGH	57	22.3	Nulliparous	clear white/yellow	16.6	18.2	22.3	24.6	20.3
**sample 146**	HIGH	60	19.8	Parous	bloody/orange/pink	19.6	20.7	24.8	27.9	23.0
**sample 147**	HIGH	58	21.6	Parous	clear white/yellow	25.4	25.5	31.7	32.9	31.8
**sample 148**	LOW	53	23.1	Parous	clear white/yellow	UD	UD	UD	UD	UD
**sample 149**	LOW	51	30.5	Parous	clear white/yellow	21.4	22.2	27.3	29.9	24.7
**sample 150**	HIGH	58	20.1	Parous	clear white/yellow	19.8	21.8	27.0	29.3	23.4
**sample 151**	HIGH	59	25.2	Nulliparous	clear white/yellow	27.1	26.5	**35.0**	**35.0**	31.8
**sample 152**	LOW	54	32.6	Parous	green/brown	26.0	25.4	**35.0**	34.0	25.6
**sample 153**	LOW	60	27.5	Parous	clear white/yellow	31.2	UD	UD	UD	32.6
**sample 154**	HIGH	56	23.2	Parous	clear white/yellow	UD	UD	UD	UD	UD
**sample 155**	HIGH	53	20.4	Parous	clear white/yellow	UD	UD	UD	34.2	34.1
**sample 156**	LOW	50	34.6	Parous	clear white/yellow	18.5	19.6	24.2	26.5	22.0
**sample 157**	LOW	57	28	Parous	bloody/orange/pink	18.7	20.7	**35.0**	27.0	21.5
**sample 158**	HIGH	65	18.6	Parous	clear white/yellow	20.5	**28.0**	26.5	29.6	24.2
**sample 159**	HIGH	59	22.8	Parous	clear white/yellow	19.9	21.3	26.8	29.5	24.4
**sample 160**	LOW	55	38.6	Parous	cloudy white/yellow	21.8	22.3	26.7	29.0	23.4
**sample 161**	HIGH	63	18.4	Nulliparous	clear white/yellow	UD	UD	UD	UD	UD
**sample 162**	LOW	53	28.3	Parous	clear white/yellow	19.1	20.3	25.5	28.9	23.7
**sample 163**	HIGH	57	24.5	Parous	clear white/yellow	18.4	19.8	24.7	27.3	21.8
**sample 164**	HIGH	71	22.1	Parous	clear white/yellow	19.9	20.8	26.7	29.6	24.6
**sample 165**	LOW	58	35.1	Parous	green/brown	23.6	23.6	28.9	30.7	25.1
**sample 166**	HIGH	56	20.7	Parous	cloudy white/yellow	27.0	26.3	31.0	34.0	28.2
**sample 167**	HIGH	51	28.7	Nulliparous	clear white/yellow	28.5	26.6	33.7	33.0	31.2
**sample 168**	LOW	59	24.5	Parous	clear white/yellow	31.4	UD	UD	UD	33.6
**sample 169**	HIGH	51	22.7	Parous	cloudy white/yellow	23.5	23.0	29.2	29.1	23.9
**sample 170**	HIGH	56	21.3	Nulliparous	cloudy white/yellow	25.2	24.7	31.2	31.0	26.7

Table S4. Baseline table comparing the discovery cohort (N=41) with the validation cohort (N=170). Significant differences are shown in bold. Cohort size per baseline characteristic can differ due to missing values. Bold p-values indicate significant difference.

		Discovery	Validation	P value
Age		N=41	N=170	0.802
	Median (range)	55 (50-72)	55 (50-74)	
BMI		N=38	N=157	0.554
	Median (range)	24.2 (18.4-38.8)	24.7 (17-49.6)	
Age at first live birth		N= 33	N=139	0.079
	Median (range)	29 (18-38)	28 (19-42)	
Age at menarche		N=41	N=162	0.626
	Median (range)	13 (9-18)	13 (9-17)	
Parity	Nulliparous (n=33)	8 (20%)	25 (15%)	0.506
	Parous (n=172)	33 (80%)	139 (85%)	
First degree BC	Yes (n=34)	8 (26%)	26 (19%)	0.427
	No (n=131)	23 (74%)	108 (81%)	
NAF colour	Clear white/yellow (n=90)	11 (27%)	79 (47%)	**< 0.0001**
	Turbid white/yellow (n=48)	3 (7%)	45 (27%)	
	Bloody/orange/pink (n=44)	13 (32%)	31 (18%)	
	Green/brown (n=28)	14 (34%)	14 (8%)	
